# Supplementary material for: Supercurrent in Bi4Te3 Topological Material-Based Three-Terminal Junctions
Source: Nanomaterials (Basel). 2023 Jan 10;13(2):293. doi: 10.3390/nano13020293 (PMC9867302; doi:10.3390/nano13020293)
Supplement: Supplementary file 1 [file nanomaterials-13-00293-s001.zip › nanomaterials-2136868-supplementary.pdf]

Supplementary Material: Supercurrent in Bi_4Te_3 Topological Material-Based Three-Terminal Junctions

Jonas Kölzer,[1,2] Abdur Rehman Jalil,[1,2] Daniel Rosenbach,[1,2] Lisa Arndt,[3] Gregor Mussler,[1,2] Peter Schüffelgen,[1,2] Detlev Grützmacher,[1,2] Hans Lüth,[1,2] and Thomas Schäpers[1,2,*]

[1] *Peter Grünberg Institut (PGI-9), Forschungszentrum Jülich, Wilhelm-Johnen-Straße, 52425 Jülich, Germany*
[2] *JARA-Fundamentals of Future Information Technology, Jülich-Aachen Research Alliance, Forschungszentrum Jülich and RWTH Aachen University, 52425 Jülich, Germany*
[3] *JARA Institute for Quantum Information, RWTH Aachen University, 52056 Aachen, Germany*

(Dated: January 10, 2023)

SUPPLEMENTARY NOTE 1: SINGLE JUNCTION MEASUREMENTS

As a reference a single Nb/Bi_4Te_3/Nb junction was measured. The junction has a length of 140 nm and a width of 500 nm. In Supplementary Figure S1a) the current voltage characteristics is shown at temperatures in the range from 30 mK to 0.77 K. At lowest temperature a critical current of 750 nA is obtained. In contrast to the three terminal junction, here, a hysteretic behaviour is observed, which can be explained by the missing shunt for the single Josephson junction. We attribute the hysteresis to heating resulting in a lower return current I_r compared to I_c [1]. The critical current monotonously decreases with temperature with some kink around 0.4 K. The latter might be attributed to a switching from a more diffusive to a more ballistic transport in the weak link [2].

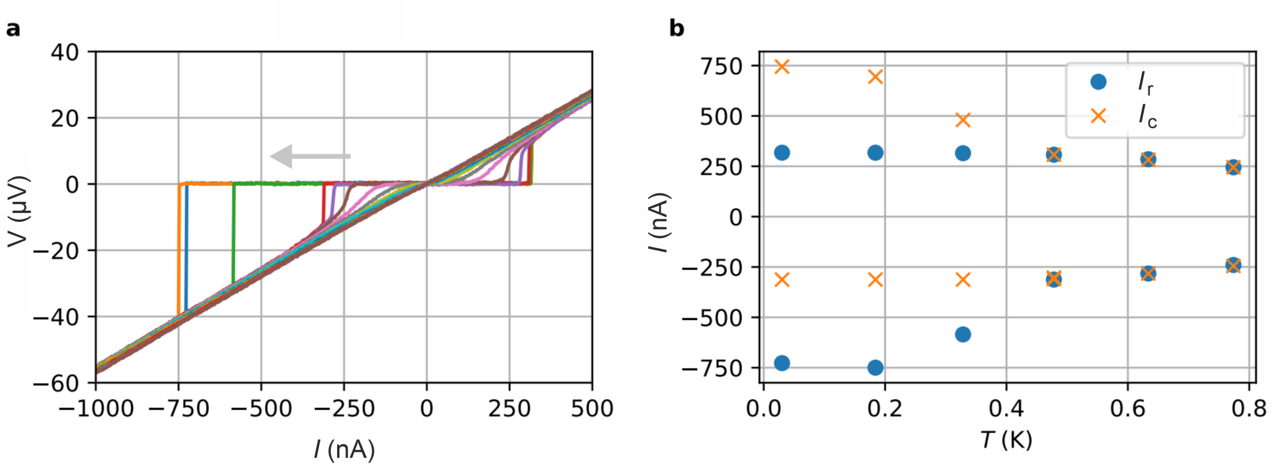

Supplementary Figure S1. Current-voltage characteristics of a single Nb/Bi_4Te_3/Nb junction: (a) Current-voltage characteristics at temperatures ranging from 30 mK to 0.77 K. (b) Critical current I_c as well as return current I_r as a function of temperature.

SUPPLEMENTARY NOTE 2: RCSJ MODEL FOR A THREE-TERMINAL JUNCTION

The characteristics of our three-terminal junctions is simulated by employing a two-dimensional resistively and capacitively shunted Josephson junction (RCSJ) Ansatz in analogy to what was presented in previous works [3, 4]. In Fig. 3a) in the main text the corresponding network is depicted including two resistively and capacitively shunted Josephson junctions with the normal state resistance R_N and the capacitance C. We assume two identical junctions

* th.schaepers@fz-juelich.de

each having a critical current of I_c. The junctions are connected by a coupling resistor R_C representing the non-superconducting junction between electrodes L and R. Following the RCSJ Ansatz the characteristics of the three-terminal junction can be described by a set of coupled differential equations of the form:

$$\frac{I_{\mathrm{LB}}}{I_c} = \sin(\varphi_{\mathrm{LB}}) + \frac{d\varphi_{\mathrm{LB}}}{d\widetilde{\tau}} + \beta_c \frac{d^2\varphi_{\mathrm{LB}}}{d\widetilde{\tau}^2} + \frac{R_N}{R_C}\left(\frac{d\varphi_{\mathrm{RB}}}{d\widetilde{\tau}} - \frac{d\varphi_{\mathrm{LB}}}{d\widetilde{\tau}}\right), \tag{S1}$$

$$\frac{I_{\mathrm{RB}}}{I_c} = \sin(\varphi_{\mathrm{RB}}) + \frac{d\varphi_{\mathrm{RB}}}{d\widetilde{\tau}} + \beta_c \frac{d^2\varphi_{\mathrm{RB}}}{d\widetilde{\tau}^2} - \frac{R_N}{R_C}\left(\frac{d\varphi_{\mathrm{RB}}}{d\widetilde{\tau}} - \frac{d\varphi_{\mathrm{LB}}}{d\widetilde{\tau}}\right), \tag{S2}$$

with φ_{LB} and φ_{RB} the phase differences between junctions J_{LB} and J_{RB}, respectively, $\widetilde{\tau} = t/\tau_J$ the normalized time, $\tau_J = \Phi_0/(2\pi I_c R_N)$, with $\Phi_0 = h/2e$ the magnetic flux quantum, and $\beta_c = (2e/\hbar) I_c R_N^2 C$ the Stewart-McCumber parameter [5]. The equations are similar to the standard RCSJ model for a single junction, except of the last term, which introduces the current through the resistor, coupling the two junctions. This current is a result of the voltage difference between the two junctions and the coupling resistance. For $R_C \to \infty$ the coupling term goes to zero, leading to two individual junctions (decoupled system) and for $R_C \to 0$ the system is dominated by the coupling term.

SUPPLEMENTARY NOTE 3: SHAPIRO STEPS IN THREE-TERMINAL JUNCTION EXPERIMENTS

The differential resistances R_{LB} and R_{RB} exposed to an rf radiation with a frequency of 5.8 GHz at 0 dBm recorded as a function of the applied DC currents are presented in Supplementary Figures S2a) and b). In contrast to the corresponding figure, which was gained by numerical differentiation, here, the resistance is directly taken using a lock-in amplifier. In Supplementary Figures S3 the corresponding measurements at a frequency of 5.8 GHz at 0 dBm are shown.

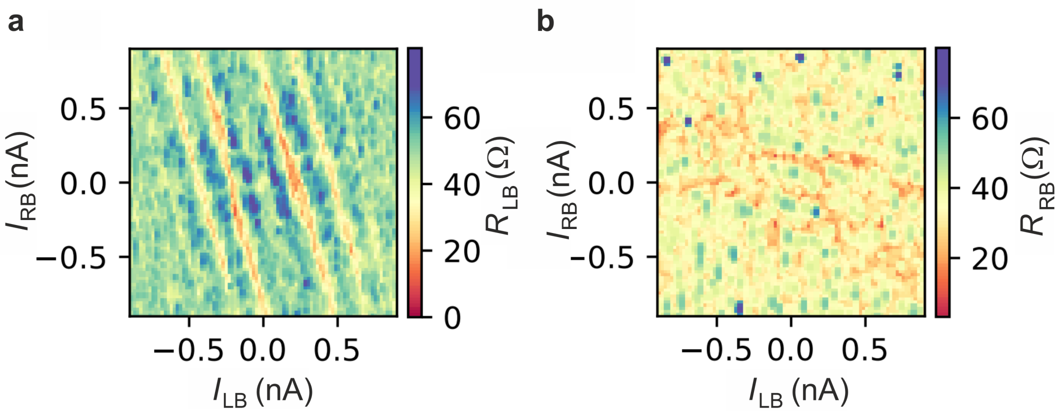

Supplementary Figure S2. Shapiro Step response at 5.8 GHz: (a) shows the measured differential resistance across the first junction R_{LB} as a function of the direct current I_{LB} and I_{RB} across the junction. (b) shows R_{RB} for the same current constellation.

SUPPLEMENTARY NOTE 4: SHAPIRO STEPS IN THREE-TERMINAL JUNCTION SIMULATION

Using the model described in Supplementary Note 2 the Shapiro response was simulated by adding an oscillation contribution $i_{\mathrm{j,rf}} \sin(2\pi f_{\mathrm{rf}} t)$, $j = \mathrm{LB}, \mathrm{RB}$, to the dc bias currents. The simulated differential resistances R_{LB} and R_{RB} as a function of the normalized voltage drops at a frequency of 8.5 GHz are presented in Supplementary Figures S4a) and b). One finds that the Shapiro response is strong in the corresponding junctions, while the coupling from the neighboring junction is weak.

In order to simulate the appearance of the fractional Shapiro steps a non-sinusoidal current-phase relationship was assumed for the Josephson junction by including a $\sin(2\varphi)$ contribution. In Supplementary Figures S5a) and b) the

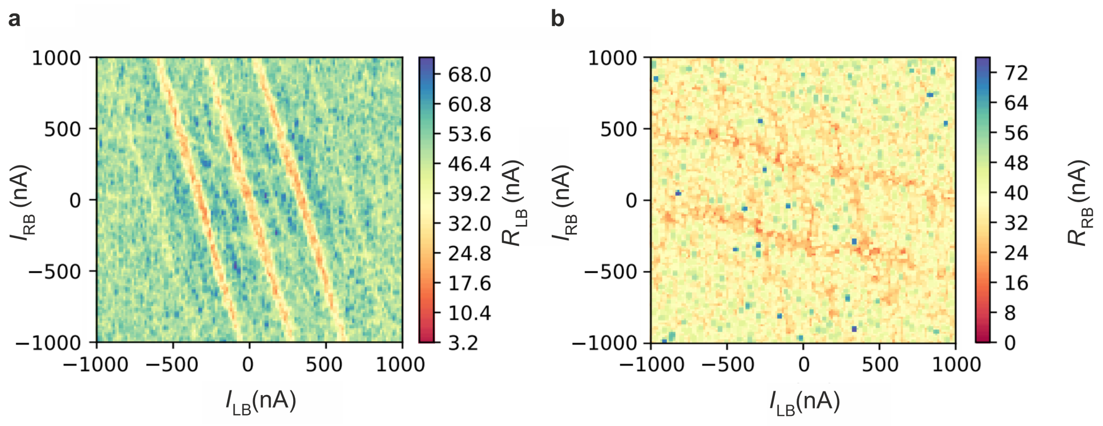

Supplementary Figure S3. Shapiro Step response at 8.5 GHz: **a** shows the measured differential resistance across the first junction R_{LB} as a function of the direct current I_{LB} and I_{RB} across the junction. **b** shows R_{RB} for the same current constellation.

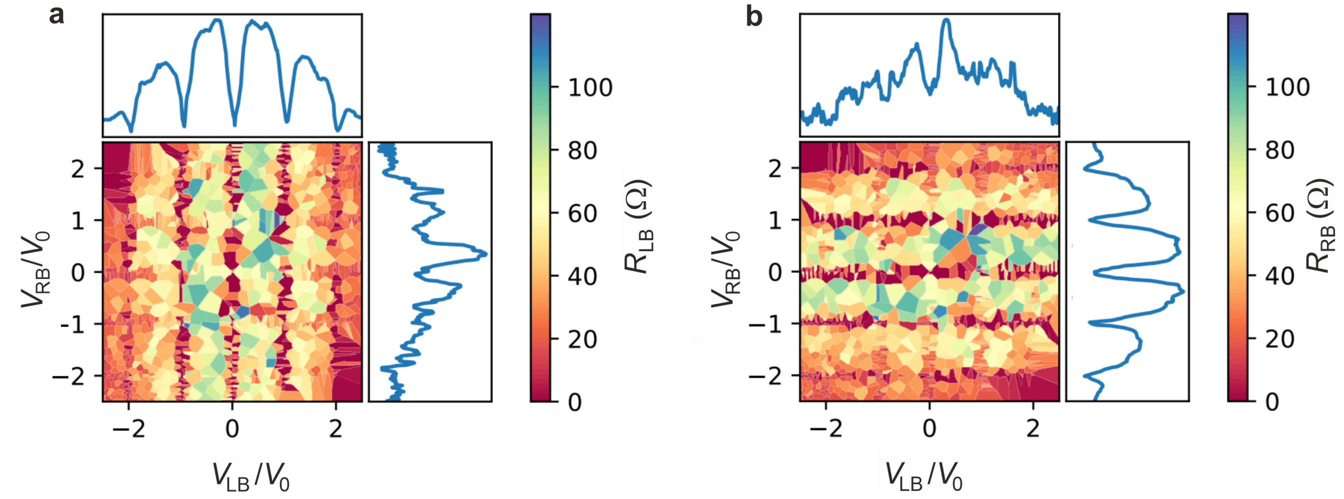

Supplementary Figure S4. Shapiro step simulations at 8.5 GHz: (a) Numerically determined differential resistance R_{LB} as a function of the normalized voltage drops V_{LB}/V_0 and V_{RB}/V_0 at 8.5 GHz. The blue curves represent the averaged signal along V_{LB}/V_0 and V_{RB}/V_0, respectively. (b) Corresponding map of the differential resistance R_{RB} with the blue curves representing the averaged differential resistance along V_{LB}/V_0 and V_{RB}/V_0, respectively.

respective simulation outcomes R_{LB} and R_{RB} for junctions J_{LB} and J_{RB} are shown as a function of bias currents. One finds that by increasing the $\sin 2\varphi$ contribution fractional steps appear.

SUPPLEMENTARY REFERENCES

[1] H. Courtois, M. Meschke, J. T. Peltonen, and J. P. Pekola, Origin of hysteresis in a proximity Josephson junction, Physical Review Letters **101**, 067002 (2008).

[2] P. Schüffelgen, D. Rosenbach, C. Li, T. W. Schmitt, M. Schleenvoigt, A. R. Jalil, S. Schmitt, J. Kölzer, M. Wang, B. Bennemann, U. Parlak, L. Kibkalo, S. Trellenkamp, T. Grap, D. Meertens, M. Luysberg, G. Mussler, E. Berenschot, N. Tas, A. A. Golubov, A. Brinkman, T. Schäpers, and D. Grützmacher, Selective area growth and stencil lithography for in situ fabricated quantum devices, Nature Nanotechnology **14**, 825 (2019).

[3] G. V. Graziano, J. S. Lee, M. Pendharkar, C. J. Palmstrøm, and V. S. Pribiag, Transport studies in a gate-tunable three-terminal josephson junction, Phys. Rev. B **101**, 054510 (2020).

[4] E. G. Arnault, T. F. Q. Larson, A. Seredinski, L. Zhao, S. Idris, A. McConnell, K. Watanabe, T. Taniguchi, I. Borzenets, F. Amet, and G. Finkelstein, Multiterminal inverse ac josephson effect, Nano Letters **21**, 9668 (2021).

[5] M. Tinkham, *Introduction to Superconductivity* (Dover Publications, New York, 2004).

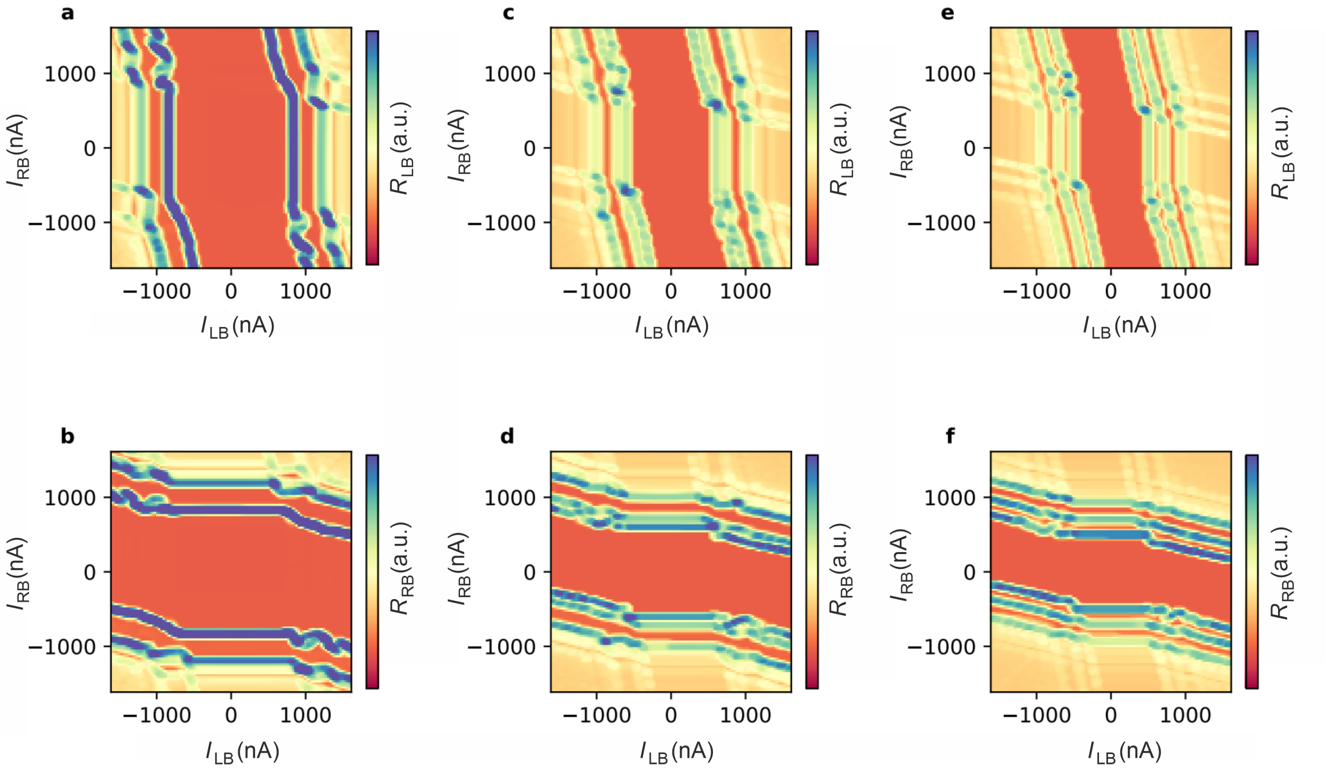

Supplementary Figure S5. Simulation of Shapiro maps at 8.5 GHz with 2ϕ term: (a) differential resistance of the first junction Shapiro steps as a function of $I_{\mathrm{LB}}, I_{\mathrm{RB}}$ with an equal contribution of a $\sin 2\phi$-term in the system, (b) the same for the second junction. (c) and (d) show the same after doubling the $\sin 2\phi$ contribution in the system and (e) and (f) show the same after doubling the contribution of (c) and (d).
